# Influence of plasma treatment on SiO_2_/Si and Si_3_N_4_/Si substrates for large-scale transfer of graphene

**DOI:** 10.1038/s41598-021-92432-4

**Published:** 2021-06-23

**Authors:** R. Lukose, M. Lisker, F. Akhtar, M. Fraschke, T. Grabolla, A. Mai, M. Lukosius

**Affiliations:** 1 IHP- Leibniz Institut für innovative Mikroelektronik, Im Technologiepark 25, 15236 Frankfurt (Oder), Germany; 2 Technical University of Applied Science Wildau, Hochschulring 1, 15745 Wildau, Germany

**Keywords:** Engineering, Materials science

## Abstract

One of the limiting factors of graphene integration into electronic, photonic, or sensing devices is the unavailability of large-scale graphene directly grown on the isolators. Therefore, it is necessary to transfer graphene from the donor growth wafers onto the isolating target wafers. In the present research, graphene was transferred from the chemical vapor deposited 200 mm Germanium/Silicon (Ge/Si) wafers onto isolating (SiO_2_/Si and Si_3_N_4_/Si) wafers by electrochemical delamination procedure, employing poly(methylmethacrylate) as an intermediate support layer. In order to influence the adhesion properties of graphene, the wettability properties of the target substrates were investigated in this study. To increase the adhesion of the graphene on the isolating surfaces, they were pre-treated with oxygen plasma prior the transfer process of graphene. The wetting contact angle measurements revealed the increase of the hydrophilicity after surface interaction with oxygen plasma, leading to improved adhesion of the graphene on 200 mm target wafers and possible proof-of-concept development of graphene-based devices in standard Si technologies.

## Introduction

One of the limiting factors of graphene integration into electronic and photonic devices is the unavailability of large-scale graphene, directly grown on isolators. Therefore, for the development of graphene-based devices, graphene transfer process from the growth wafer (donor) onto the isolating (target) wafer needs to be performed. Different graphene transfer methods have been described in literature, which can be classified as dry^1–6^ or wet^7–9^ transfer methods depending on the environment the graphene is touching the target wafer during the transfer procedure. Dry graphene transfer is based on the employment of the intermediate polymer layer (e. g. PDMS stamp^10^) or by usage of release tape^11^ to avoid the direct contact of graphene with the wet solution; however, this method suffers from polymer residuals and defects like cracks, folds, and wrinkles after the transfer procedure due to the strong graphene-substrate interaction. In order to weaken it, several treatments have been investigated in the literature to ease the release of graphene from the donor wafer. Intercalation at the graphene-metal interface has also been studied in order to tune the interaction strength and to oxidize the metal surface below graphene before the graphene transfer process. For example, water intercalation^12^ process, where water penetrates into the graphene-metal interface for easier graphene transfer from the donor to target wafer was reported. The disadvantage of such a method is long interaction times (16 h to up to 3 days) and low process control for wafer-scale graphene transfer. On the other hand, the intercalation with carbon monoxide^13^ or chemical modification^14^ of the graphene-metal interface to achieve higher transfer speeds has also been reported in the literature.

Another group of graphene transfer methods is based on chemical etching of the catalytic substrates on which graphene is grown. The main problem of chemical etching is metal cross-contamination, which alters the electrical properties of the transferred grapheme^15^. The alternative method of chemical etching is the wet electrical delamination process, where graphene is released from various growth substrates^8,16^. This method is based on the water electrolysis process, where H_2_ formation at the Gr-metal interface detaches graphene from the growth wafer^8,16,17^ and is a faster method compared to the chemical etching process. However, the electrical delamination process also involves chemicals (e.g., NaOH) and poly(methyl methacrylate) (PMMA) as a support layer for graphene transfer procedure; therefore, the precise cleaning and polymer removal steps have to be understood and optimized in order to prevent the extrinsic doping of graphene. The optimized delamination process itself does not guarantee the successful transfer process, as surface contamination, hydrophilicity and roughness of target wafers also may influence the adhesion properties and respectively quality of transferred graphene.

Indeed, the 2D nature of graphene, where the ratio between the surface area to volume area is increased, leads to strong surface interactions affecting the adhesion capability compared with 3D materials. S. Das et al. reported the adhesion energy increase after thermal annealing procedures to improve the adhesion of graphene on SiO_x_^18^. At this point, the hydrophilicity-hydrophobicity of the target wafers is one of the most critical parameters for the adhesion of 2D graphene; however, only very few literature works deal with this phenomenon. Kim et al.^19^ showed the relation between the SiO_2_ surface wettability and graphene quality transferred by wet transfer to reduce the content of wrinkles in graphene after the transfer on the target wafer. However, the organic solvent (instead of DI water) was used in their work, making the transfer process more complex. Another study involved aluminum nitride (AlN) surfaces, as they were investigated as a target wafers for graphene transfer^20^. Different surface treatments lead to increased surface wettability as detected by measured wetting contact angle (WCA) measurements between solid surface and water drop. This clearly shows the need for further investigations and understandings of the relation between surface wettability properties and therefore the adhesion of graphene on the target wafers.

In this paper, graphene was transferred by the electrochemical delamination process from 200 mm Ge/Si donor wafers on 200 mm, Si technology standard SiO_2_/Si and Si_3_N_4_/Si target wafers by changing their hydrophilic behavior with an additional surface treatment to achieve low-defect and up-scaled graphene transfer process. The electrochemical delamination process was selected since wafer-scale graphene transfer through wafer bonding or etching of Ge under graphene is still not achieved for Gr/Ge/Si system. In the graphene growth-transfer process, no metals (e.g., Cu, Ni, etc.) were involved; therefore, metal cross-contamination problems were avoided. In the present research, the surface wetting contact angle measurements of the target wafers and surface composition and roughness measurements by XPS and AFM were performed, respectively. The obtained results allowed us to determine the relationship between surface composition, roughness, wettability, and graphene’s adhesion ability on the target wafers with respect to additional surface treatment by oxygen plasma. Finally, the improved graphene adhesion was achieved for possible future fabrication of proof-of-concept graphene-based devices in standard Si technologies.

## Results and discussion

In the present research, four different target surfaces were selected for the transfer experiments of graphene. Three types of SiO_2_ (HDP- SiO_2_, TEOS—SiO_2_, and thermal—SiO_2_) and one type of Si_3_N_4_ (PE-Si_3_N_4_) films were employed as target surfaces on standard Si wafers. To be precise, HDP stands for high-density plasma deposition using silane (SiH_4_) precursor at 650 °C deposition temperature, TEOS is the deposition of SiO_2_ by using the tetraethyl(ortho)silicate (TEOS) precursor and plasma at 400 °C, whereas thermal SiO_2_ is produced at a temperature of 1000 °C. Si_3_N_4_ films were grown by plasma enhanced (PE) chemical vapor deposition at 400 °C by using silane and NH_3_/N_2_ gasses as precursors. For all SiO_2_ and Si_3_N_4_ target surfaces the graphene transfer and post-transfer processing steps were identical in order to observe the effect of target surface and dependences with respect to certain changes.

**Figure 1:**
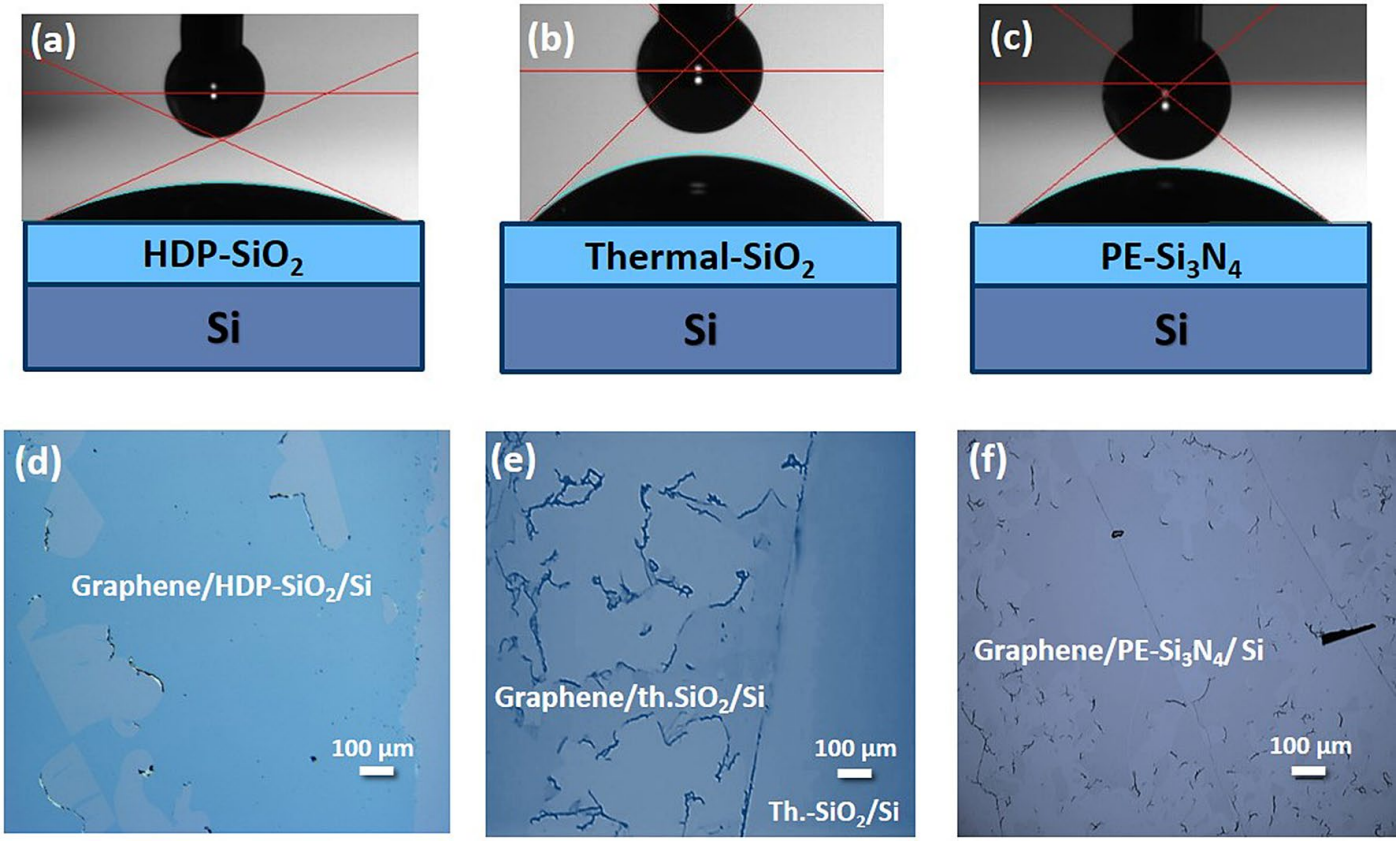
Representative pictures of wetting contact angles of (**a**) HDP-SiO_2_ with WCA = 31°, (**b**) Thermal-SiO_2_ with WCA = 45°, (**c**) PE-Si_3_N_4_ with WCA = 38° film surfaces and optical microscopy pictures of graphene flakes transferred on corresponding surfaces (**d**) HDP-SiO_2_ (**e**) Thermal-SiO_2_, (**f**) PE-Si_3_N_4_.

Firstly, the chemical affinity/surface reactivity of SiO_2_, Si_3_N_4_ target surfaces was investigated by measuring the wettability contact angle (WCA) with the sessile drop method^21^. By this method, the interaction and spreading of a small volume (1µL) water droplet on a solid surface is investigated. The contact angle (θ) for a flat surface is described by Young equation^22^ cos (θ) = (γ_SV_−γ_SL_)/γ_LV_, where (γ) is the surface tension of solid–vapor (SV), solid–liquid (SL) and liquid–vapor (LV) interfaces, respectively. Theoretically, surface is hydrophilic if the contact angle between the solid surface and water is below 90°. On the hydrophilic surfaces, water tends to spread out on the material surface due to higher adsorption as binding energies between water molecules. The observed contact angles revealed the hydrophilic behavior of SiO_2_ and Si_3_N_4_ surfaces independently on the deposition conditions—contact angles were below 90° (Fig. 1a–c). The as-grown 100 nm SiO_2_ films had contact angles of 25°, 31°, and 45° for HDP- SiO_2_, TEOS—SiO_2_, and thermal—SiO_2_, respectively. The observed WCA values for SiO_2_ agrees with data reported in the literature, where the WCA varies from ca. 28° to 52°^19,20,23^. The WCA for as-grown 40 nm thick PE-Si_3_N_4_ films were approximately equal to 38°.

Despite the hydrophilic nature of SiO_2_ and Si_3_N_4_ layers, the transfer experiments on the described target wafers resulted in mechanically damaged graphene (Fig. 1d–f). The achieved quality of transferred graphene flakes was not suitable for further development of large-scale graphene-based devices; therefore, further experiments were performed to solve the observed transfer challenges.

**Figure 2:**
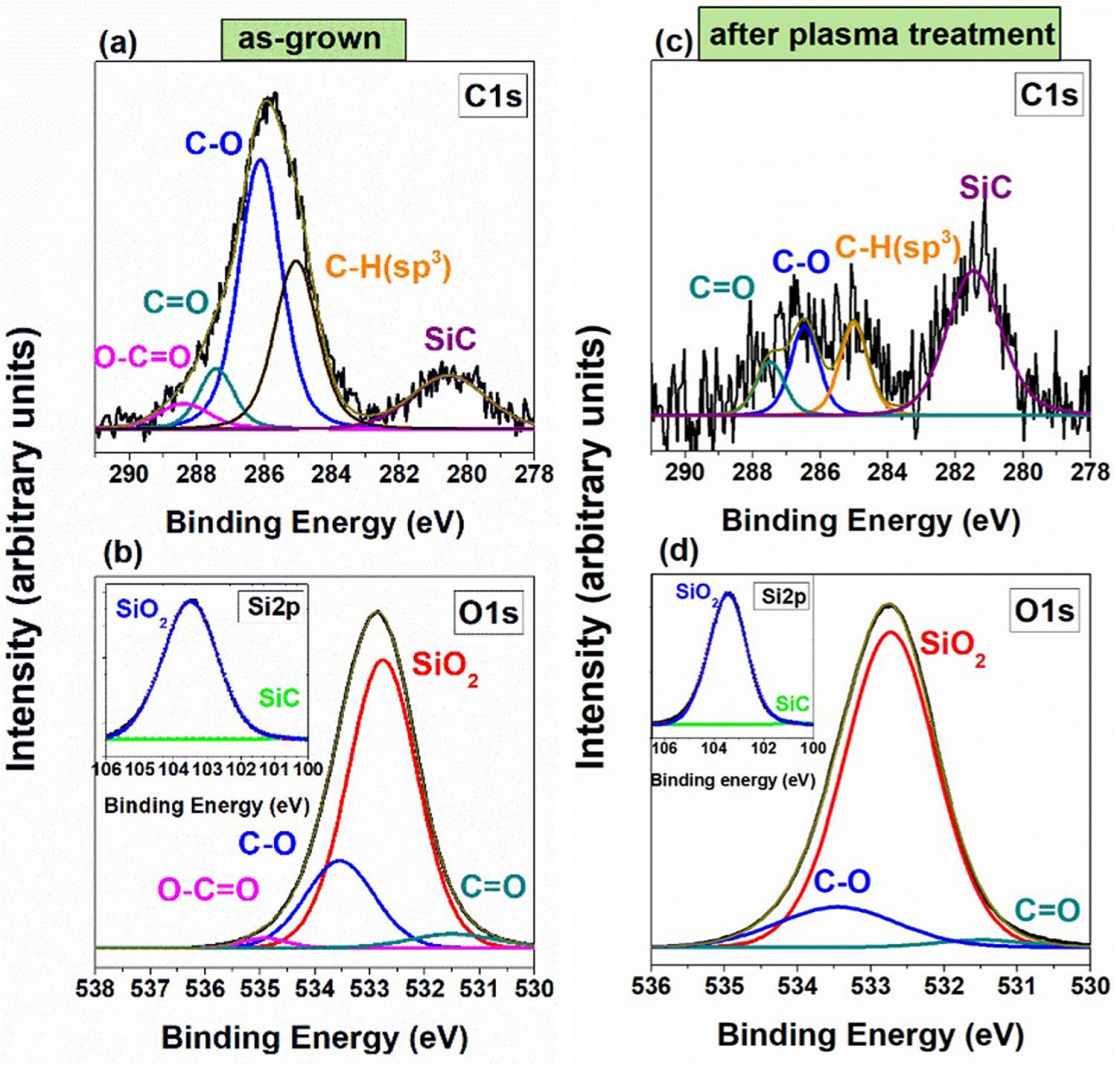
The XPS spectra of HDP-SiO_2_ oxide (**a**) C 1s spectra, (**b**) O1s spectra, (**b inset**) – Si 2p spectra—for as-grown HDP oxide; (**c**) C 1s spectra (**d**) O 1s spectra (**b inset**)—for plasma treated HDP- SiO_2_ oxide.

To determine if surface contamination of target wafers could be the reason for inhomogeneous graphene flake adhesion, the XPS spectroscopy measurements were performed firstly for SiO_2_ and lately for Si_3_N_4_ surfaces. The fitting of the C 1s peak at 286 eV revealed the characteristic peaks related to carbon-containing groups C–H, C–O, C = O, –O–C = O (Fig. 2a). This tendency was noticed for all SiO_2_ films; however, in the case of thermal-SiO_2,_ the surface contamination by carbon-containing groups is lower. No functional ester group (–O–C = O) was detected, related to high process temperature (1000 °C) and therefore higher stability towards adsorption from the ambient (SI Fig. S1). Despite the different precursors and process parameters for the deposition of SiO_2_ films, the surface contamination was quite similar for all surfaces of investigated oxide films. Additionally, the very clear and intensive peak at the binding energies of 280.7 eV was observed, which was assigned to Si–C bonding. Similar surface contamination tendency was observed from the fitting of O 1s peak at 532.8 eV binding energy (Fig. 2b). The main peak is attributed to SiO_2_, however also in O1s spectra, carbon-containing organic groups were observed at the surface of the samples, including the above-mentioned C–O, C=O, –O–C=O groups for HDP- and TEOS-SiO_2_ (SI Fig. S1). Whereas, for thermal-SiO_2_, –O–C=O functional group was not observed on the surface, due to the same reasons as mentioned above in the explanation for C1s spectra.

No differences in Si 2p spectra were noticed for differently grown SiO_2_ films, where mainly the characteristic Si–O peak at a binding energy of 103.5 eV was detected (Fig. 2b inset and SI Fig. S1b, d, f insets). In order to reduce the content of C-containing groups on the surface of SiO_2_ films, the oxygen plasma treatment was applied. The XPS analysis on plasma treated SiO_2_/Si and Si_3_N_4_/Si surfaces revealed surface composition changes compared to as-grown SiO_2_ films (Fig. 2c, d, and SI Fig. S2). For oxides, fitting of characteristic C 1s peak at 286 eV revealed the obvious reduction of corresponding C–H, C–O, –O–C=O bond peaks after plasma treatment and was mainly related to the reduction of surface contamination (Fig. 2c, and SI Fig. S2a, c, e). The functional –O–C=O group was completely removed from the surface of HDP-SiO_2_ films after the interaction with oxygen plasma (Fig. 2c). For the surface of TEOS-SiO_2_ films, the C1s spectra showed reduced surface contamination after plasma treatment; however, still containing all surface contaminants as before plasma treatment (SI Fig. S2c). The elongated exposer times for TEOS-SiO_2_ films should be applied for getting cleaner surfaces; however, the precise exposer time control is required to avoid the damaging or (and) doping of the surface. In the case of thermal-SiO_2_, the functional –C=O group was completely removed from the surface after plasma treatment (SI Fig. S2e). The observed Si–C peak at binding energies of 280.7 eV was not affected by plasma treatment for all SiO_2_ films. The fitting of the O 1s region in XPS spectra after plasma treatment (Fig. 2d and S2) showed exactly the same behavior as in the C 1s spectra. Additionally to the main SiO_2_ peak at 532.8 eV binding energy, carbon-containing groups were detected on as-grown SiO_2_ surfaces, whereas the exposer to oxygen plasma leads to a reduction of carbon-containing groups and removal of –O–C=O group for HDP-SiO_2_, and –C=O group for thermal-SiO_2_ (Fig. 2d and S2f.). In the case of TEOS-SiO_2,_ all functional C-containing groups remained on the surface, as detected in C 1s spectra (Fig. S2d). To conclude, thermal SiO_2_ was produced at a higher process temperature (1000 °C), and it seems that the surface of thermal oxide is more stable compared with other oxides, leading to less C-contaminated surface and lower surface modification after oxygen plasma treatment at 250 °C. However, the plasma effect on HDP- and TEOS-SiO_2_ surfaces were rather similar and revealed a slight reduction in surface contamination.

**Figure 3:**
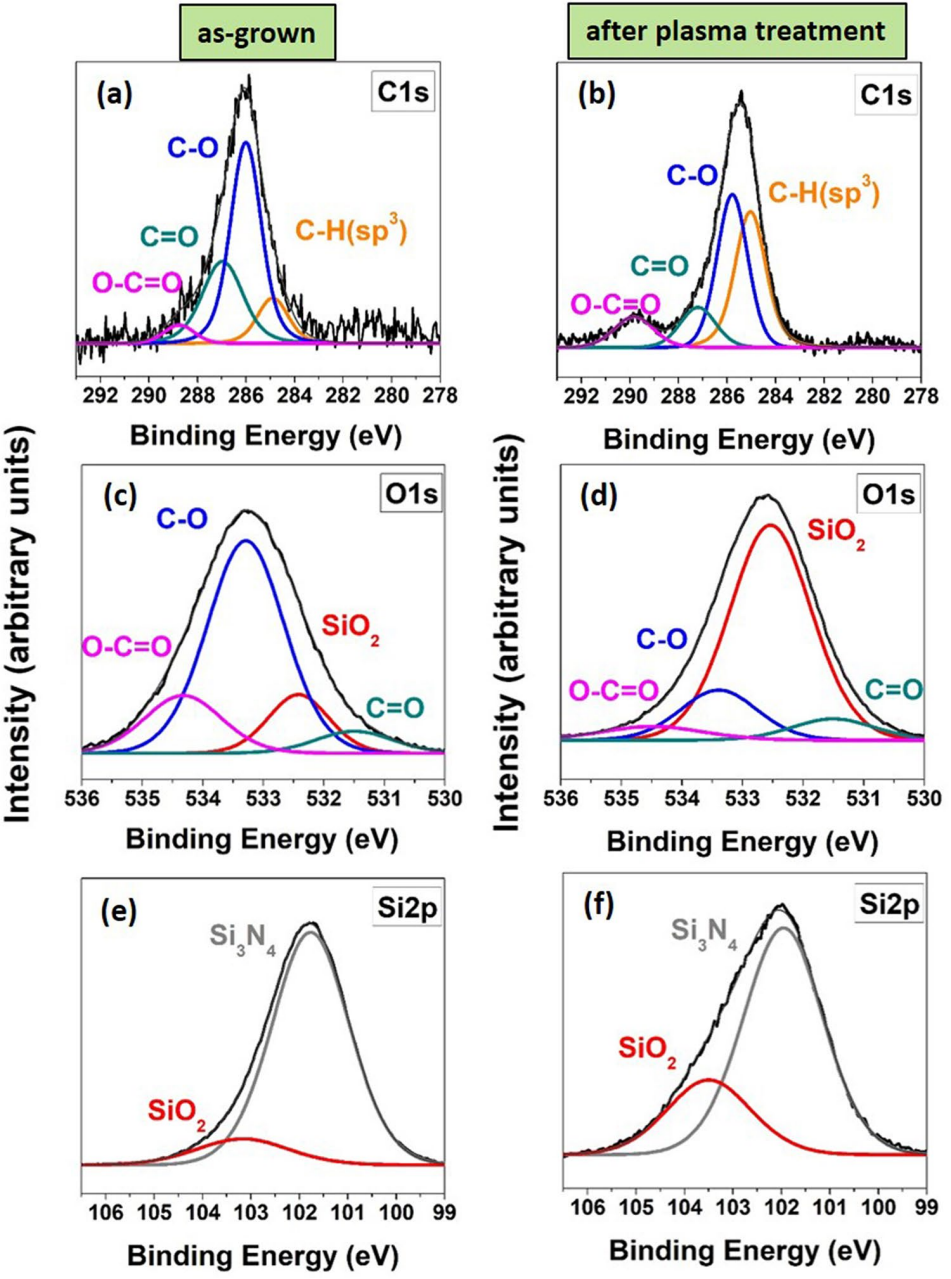
The characteristic XPS spectra of PE-Si_3_N_4_ surface (**a**) C 1s spectra for as-grown**,** (**b**) C 1s spectra for plasma treated, (**c**) O 1 s spectra for as-grown, (**d**) O 1s spectra for plasma treated**,** (**e**) Si 2p spectra for as-grown (**f**) Si 2p spectra for plasma treated.

The XPS analysis was also performed for as-grown and plasma treated Si_3_N_4_/Si surface. In the characteristic C1s spectra, higher C–O peak was observed for as-grown S_3_N_4_ films (Fig. 3a) in comparison to plasma treated surface of S_3_N_4_ (Fig. 3b). Correspondingly, in the O1s spectra the intensive C–O spectra was reduced and increase of SiO_2_ peak was observed if the surface of as-grown S_3_N_4_ films (Fig. 3c) was treated with oxygen plasma (Fig. 3d). The slight shift in C1s and O1s spectra towards lower binding energies were observed for plasma treated surface and is related to reduction of C–O components. In Si2p spectra characteristic SiO_2_ peak at 103.5 eV binding energy (Fig. 3e) also increased for the plasma treated S_3_N_4_ surface (Fig. 3f). In the Si2p spectra, the intensity of the Si–O peak increases in respect to the Si–N peak, and broadening of the Si 2p (Fig. 3f) is probably due to partial exchange of N by O during the interaction of Si_3_N_4_ with plasma generated O radicals. Luhmann et al.^24^ also observed (from XPS) the substitution of oxygen and depletion of nitrogen at the surface of LP-CVD silicon nitride films after the exposer to oxygen plasma. Like in the SiO_2_ case, the plasma treatment leads to a slight reduction of C-contamination on the film surface (Fig. 3b), whereas the fitting of N1s spectra before and after plasma treatment revealed no obvious peak intensity and composition changes (SI Fig. S3).

**Figure 4:**
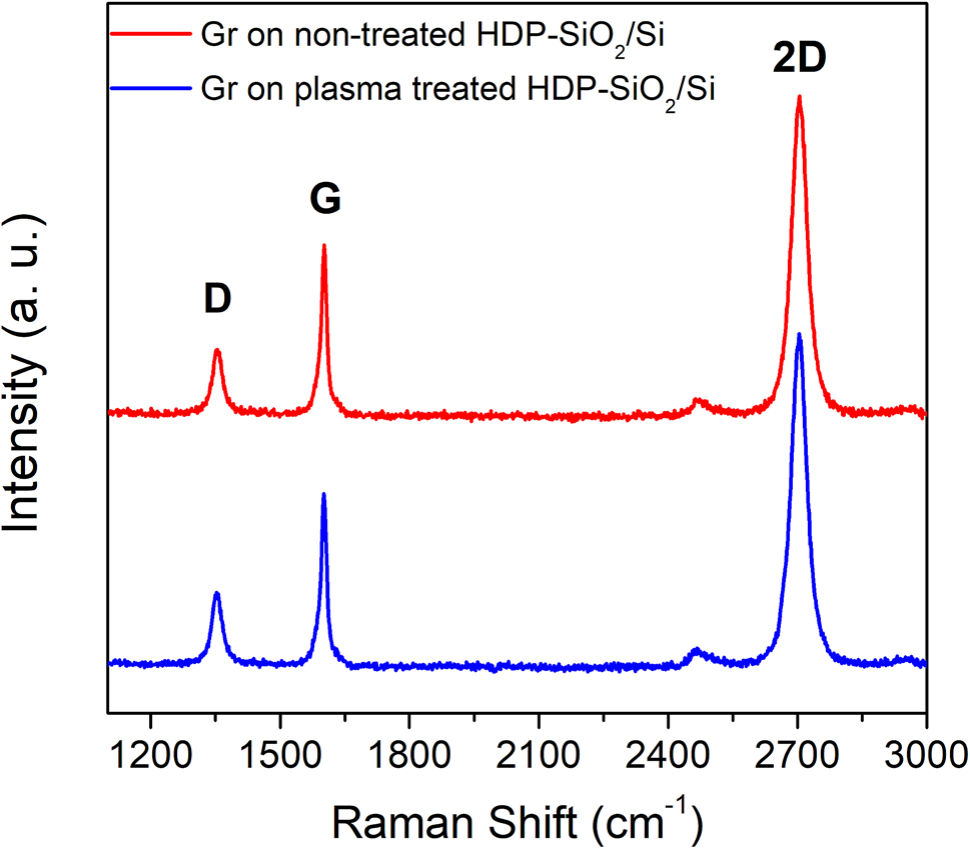
Raman spectra for graphene transferred on plasma treated HDP-SiO_2_/Si (blue curve) and on non-treated HDP-SiO_2_/Si wafer (red curve).

Additionally, the XPS analysis was performed to determine if Ge cross-contamination and electrolyte residuals (NaOH) appeared after the graphene growth and transfer on the target wafer, respectively. No characteristic Na 1s and Ge 2p peaks were observed in XPS spectra for all investigated transferred graphene samples (SI Fig. S4), or the contamination level was below the detection limit. Furthermore, the possible graphene doping through the oxygen plasma treatment of target wafers was analyzed by Raman spectroscopy. No shifts in G and 2D peaks of graphene have been observed on the samples, transferred on plasma treated SiO_2_ and the non-treated samples (Fig. 4. for HDP-SiO_2_/Si). No change of 2D/G (~ 1.8) ratio was noticed for plasma treated surfaces, indicating no doping or quality reduction after plasma treatment of investigated surfaces before graphene transfer.

**Figure 5:**
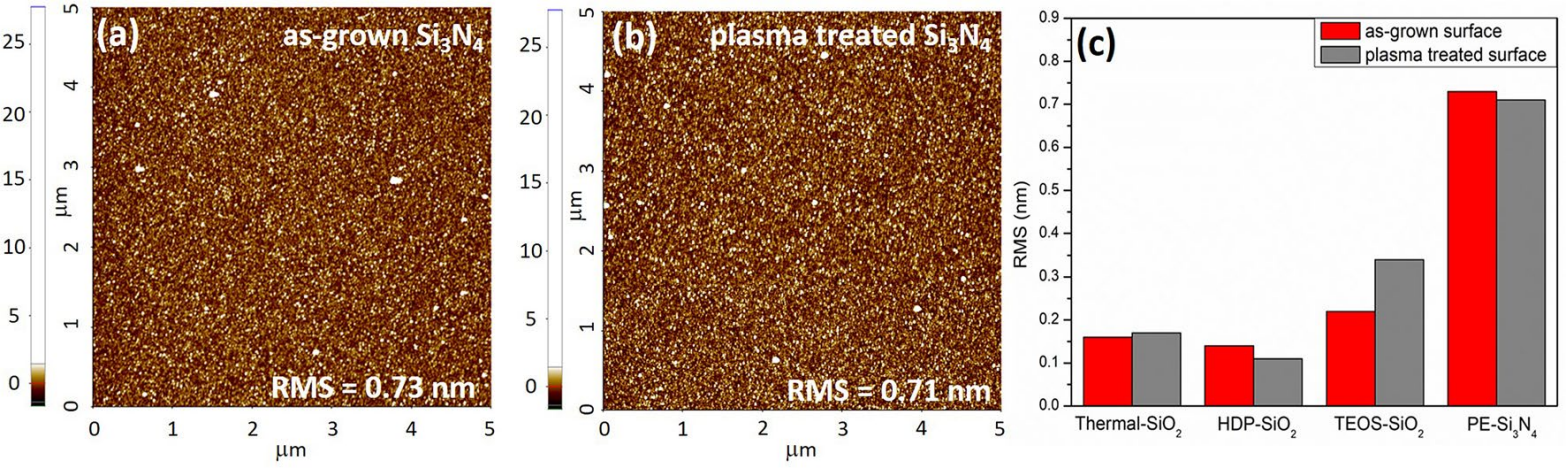
Surface morphology (5 × 5 µm) of PE-Si_3_N_4_ films measured by Atomic Force Microscopy: (**a**) as-grown PE-Si_3_N_4_, (**b**) PE-Si_3_N_4_ after oxygen plasma treatment, (**c**) comparison of Root-mean-square (RMS) surface roughness for as-grown and plasma treated SiO_2_ and Si_3_N_4_ surfaces.

Furthermore, the possible plasma effect on surface roughness was analyzed by atomic force microscopy for all SiO_2_/Si and Si_3_N_4_/Si surfaces. The as-grown SiO_2_ films revealed very low surface roughness (RMS) equal to 0.14 ± 0.02, 0.17 ± 0.02, and 0.22 ± 0.04 nm for HDP-SiO_2_, thermal-SiO_2_, TEOS-SiO_2_ films, respectively. In literature, the RMS values vary from 0.4 to 1.9 nm for as-grown oxide films^20,25,26^, depending on the deposition method. The surface roughness of as-grown Si_3_N_4_ films was equal to 0.73 ± 0.06 nm (Fig. 5a); however, no obvious difference in the quality of transferred graphene was noticed in comparison to graphene transfer experiments on SiO_2_ shown in Fig. 1. Whereas Knapp et al.^20^ observed the reduction of surface roughness for plasma-treated AlN surfaces, in the present research, no obvious reduction of surface roughness was observed for Si_3_N_4_ (Fig. 5). Only minor surface roughness variation before and after plasma treatment was noticed (Fig. 5c) for CVD grown SiO_2_ film as well. According to Wenzel et al.^27^ the contact angle for rough surfaces should be extracted by including the surface roughness component [cos θ_m_ = r × cos θ]. However, in the present research, no obvious roughness and morphology changes were observed for SiO_2_/Si and Si_3_N_4_/Si surfaces; therefore, the film roughness effect on an absolute value of contact wetting angle was neglected for as-grown and plasma-treated target surfaces.

**Figure 6:**
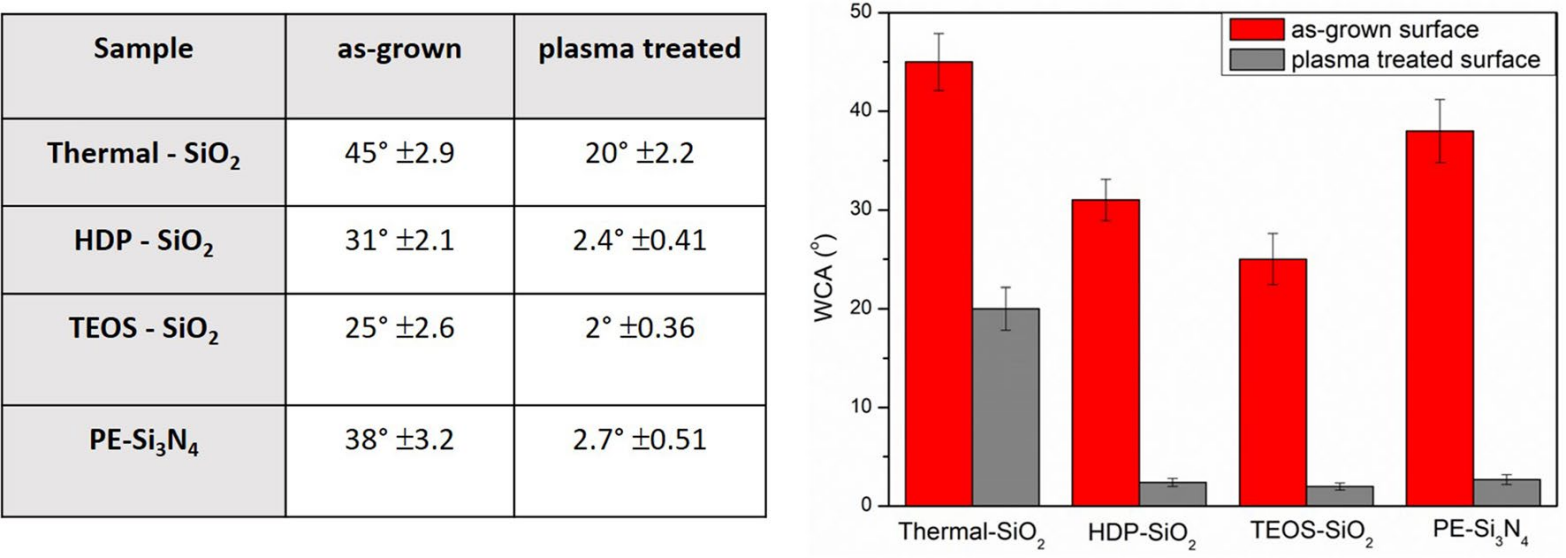
Wetting contact angle values for as-grown and plasma treated SiO_2_ and Si_3_N_4_ surfaces.

Finally, the WCA measurements have been performed on the plasma treated samples. It was indeed found that the surface hydrophilicity and, therefore, surface reactivity increased. The measured WCA angles for plasma-treated surfaces revealed the reduced wetting contact angles (Fig. 6), which is due to the surface purification after processing with oxygen plasma. During the plasma process most of organic bonds (i.e., C–H, C–C, C = C, C–O, C–N) of surface contaminants are broken and oxidized leading to formation of H_2_O, CO, CO_2_ et al. which are pumped out from the reaction chamber during the processing.

The measured WCA was equal to 2.4°, 2°, and 2.7° degrees for HDP-, TEOS-SiO_2_ and PE-Si_3_N_4_ films, respectively, resulting in highly hydrophilic surfaces. The determined WCA angle of 20° for thermal- SiO_2_ agrees with obtained XPS results (slightly affected surface composition) and confirms the relative surface stability towards oxygen plasma.

**Figure 7:**
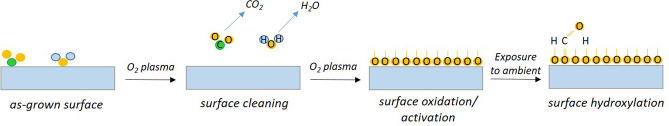
Schematic representation of possible surface modification after oxygen plasma and exposure to ambient.

According to the obtained experimental results, a simple graphical explanation, as possible surface interaction and modification model after interaction with oxygen plasma, is visualized in Fig. 7. Firstly, by oxygen plasma treatment, the C-contamination is partly removed from the investigated SiO_2_ and Si_3_N_4_ surfaces through bombardment with active radicals and particles generated through oxygen plasma (like i.e. O_2_^+^, O_2_^−^, O_3_, O, O^+^, O^−^ and free electrons). Secondly, in the case of Si_3_N_4_, oxygen from the plasma substitutes and exchanges nitrogen in Si_3_N_4_ films, leading to the partially oxidized surfaces. As determined by XPS characterization, oxygen plasma treatment leads to surface cleaning and activation, resulting in increased surface reactivity. The surface contamination reduction resulted in the increased number of dangling bonds on the surface and, therefore, higher surface energy in comparison with as-grown surfaces, increasing surface hydrophilicity of investigated SiO_2_ and Si_3_N_4_ surfaces. With increased target surface hydrophilicity the interaction between graphene carbon atoms and the target wafer atoms increases through van der Waals bonding.

**Figure 8:**
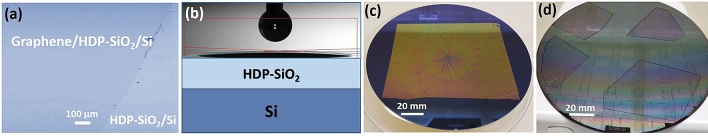
Optical microscopy picture of (**a**) graphene flake transferred on HDP-SiO_2_ surface with wettability angle of 2.4°, (**b**) schematic drawing of water drop on the surface with WCA = 2.4°, (**c**) large-scale PMMA/graphene flake transferred on HDP-SiO_2_/Si surface (**d**) transferred graphene flakes (without PMMA resist) on structured 200 mm wafer.

In relation to the performed experiments, characterization of target wafers, and the proposed interaction mechanism between reactive plasma particles and target wafer, the improved adhesion of the graphene was achieved. Due to attractive Van der Waals forces between graphene flake and target wafer surface, the graphene flakes adhered easier and more homogenously on the plasma treated target wafers prior transfer procedure, resulting in continuous and wrinkle-free transferred graphene flakes (Fig. 8, Fig. S6). Additionally, smooth surface of target wafers (Fig. 5) supported the conformal graphene adhesion in relation with increased surface reactivity through hydrophilicity increase of target wafers, due to graphene’s ability to bend in out-of-plane and to stretch in in-plane directions. The hydrophilicity increase and correspondingly reduced contact wetting angle lead to easier water escape from graphene-target wafer interface. Due to polar nature of the water its surface energy is high, therefore by treating the target wafers with plasma the surface energy increases as well, favoring the escape of water without causing the mechanical damage in transferred graphene after electrochemical delamination procedure. In Fig. 8, the improved transfer of graphene on HDP-SiO_2_ surface is presented; however, the same results were obtained for low WCA plasma-treated TEOS-SiO_2_, PE-Si_3_N_4_ surfaces. The ability to transfer graphene on the blanket and structured 200 mm wafers (Fig. 8c, d) was achieved through target wafer surface cleaning/activation by a plasma process. Additional large-scale transfer examples are presented in Fig. S5 (SI).

At this point, it is worth mentioning that other transfer-related parameters, like delamination speed, the thickness of PMMA, the post-annealing temperature and rate, the PMMA removal procedure etc., have a certain influence on the complete transfer process of graphene and had been optimized in this study as well. However, these parameters do not impact the surface hydrophilicity and enhanced wettability compared to plasma treatment procedure.

The optimized conditions are described in more detail in the experimental part.

## Methods

The SiO_2_ and Si_3_N_4_ films were deposited by CVD techniques on 200 mm Si wafers and were employed as target wafers for transferring graphene. For the deposition of SiO_2_ films, high-density plasma (HDP) and plasma-enhanced (PE) CVD processes were developed, allowing depositions also at low temperatures (below 400 °C) suitable for back-end-of-line (BEOL) in integrated circuits (IC) fabrication. Four corresponding target surfaces (HDP- SiO_2_, TEOS—SiO_2_, thermal—SiO_2_, PE- Si_3_N_4_) were selected for transfer experiments of graphene. HDP-SiO_2_ layers were deposited at 650 °C deposition temperature using SiH_4_ as precursor. TEOS-SiO_2_ films deposited at 400 °C by PE-CVD using tetraethyl(ortho)silicate (TEOS) precursor. Thermal-SiO_2_ was produced at a temperature of 1000 °C. The Si_3_N_4_ films were grown by PE-CVD at 400 °C using silane and NH_3_/N_2_ gasses as precursors. The thickness of SiO_2_ films was measured to be 100 nm, whereas Si_3_N_4_ films were 40 nm thick. The wetting contact angle (WCA) measurements to determine hydrophilicity of SiO_2_/Si, Si_3_N_4_/Si surfaces were performed by the “Surftens Automatik” technique at 20 °C temperature. The 49-point measurements were performed pro wafer and 4–6 wafers were investigated for each of investigated as-grown and plasma treated wafer surface. The oxide and nitride surfaces were treated with oxygen plasma for 25 s at 250 °C at 900 W in order to reduce the WCA. The roughness of the target surfaces was investigated by Park Systems NX20 atomic force microscopy (AFM) in the non-contact mode by using the high aspect ratio silicon tips (AR5-NCHR 10 M), operating at 330 kHz resonance frequency and having high operation stability with outstanding sensitivity and fast scanning ability. The surface composition/contamination of SiO_2_ and Si_3_N_4_ films were investigated by XPS (Physical Electronics Instruments) analysis. After deposition or/and transfer processes, the samples for XPS analysis were taken to the XPS measurement chamber within 20 min. Before starting the measurements, samples were annealed in 10^–7^ vacuum for 20 min. Raman measurements were performed by Renishaw system with a 532 nm Nd:YAG laser, 100X objective and a laser spot size of 1 µm.

Prior to the deposition of graphene, 2 µm thick Ge(100) films were grown epitaxially on 200 mm Si(100) wafers^28^. Afterward, the monolayer graphene was grown on 200 mm Ge/Si wafers by CVD technique (Aixtron Black Magic BM300T CVD) at 885 °C temperature and 700 mbar pressure, using CH_4_ and Ar/H_2_ as a source and carrier gasses, respectively^29^. The poly(methyl methacrylate) (PMMA) polymer was spin-coated on top of the grown graphene to enable and support the graphene during the wet transfer procedure. For the spin coating the E-Beam Resist PMMA 950 K (Allresist) was used and coated with 4000 rpm rotation speed resulting in ca. 400 nm thickness of PMMA on the Gr/Ge/Si stacking.

**Figure 9:**
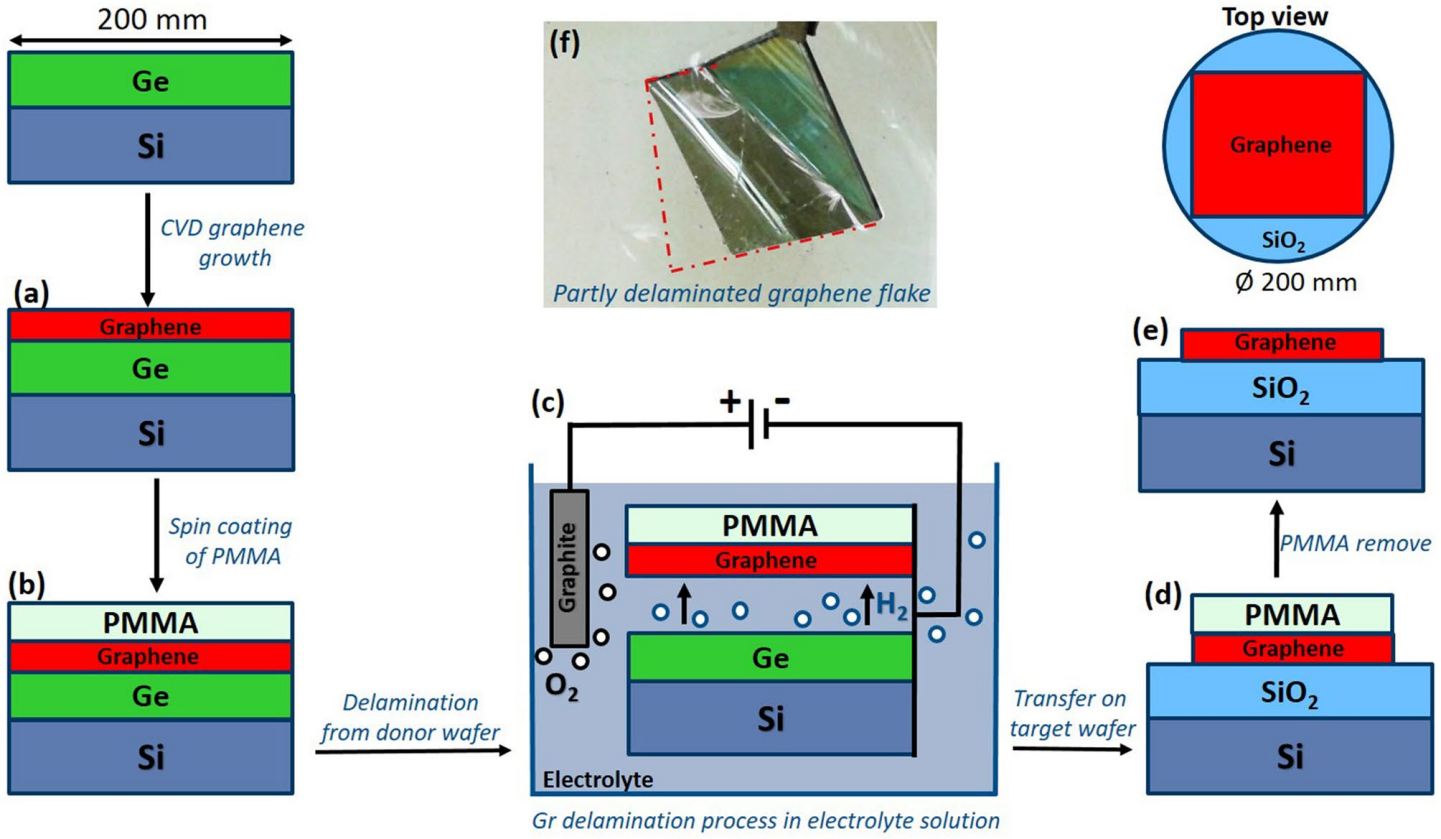
General graphene growth and transfer scheme: (**a**) graphene growth on 200 mm Ge/Si wafers by CVD process; (**b**) spin-coating of PMMA polymer on top of graphene; (**c**) electrochemical delamination process in order to detach graphene from the donor wafer; (**d**) transfer of detached graphene flake on target wafer; (**e**) transferred graphene on target wafer after the remove of support PMMA layer: cross-section and top view; (**f**) image of partly delaminated graphene flake during the delamination process.

The 200 mm wafers with PMMA/Gr/Ge/Si were cut into different pieces in the sizes from 4 × 8 cm till 13 × 13 cm before the delamination process. The size of transferred graphene flake of 13 × 13 cm is only limited by our equipment, not graphene itself, therefore full 200 mm wafer covered with graphene can be transferred by electrochemical delamination process. PMMA/graphene stack was delaminated from donor Ge/Si wafer by electrochemical delamination process in NaOH electrolyte solution, where the PMMA/graphene/Ge/Si was used as cathode and graphite plate as anode (Fig. 9) in the electrolytic cell. The used voltage varies depend on the size of the flakes and varied correspondingly from 3–12 V, for small and larger flakes respectively. The delamination time is also dependent on the graphene flake size and on the applied voltage, therefore varied from 4 to 30 s. The applied voltages varied in few volt range, depending on the size of the graphene flake: larger graphene flake—higher voltage is required. The graphene is mechanically separated from the Ge/Si wafer by H_2_ formation on the cathode (PMMA/graphene/Ge/Si) as the results of water electrolysis process. The delaminated PMMA/graphene stack was transferred onto isolating SiO_2_/Si and Si_3_N_4_/Si substrates. Post-transfer processing of the transferred graphene (PMMA/graphene/SiO_2_(Si_3_N_4_)/Si) was performed at 135 °C temperature for about 13 h in an inert atmosphere for water elimination from graphene-target wafer interface. The PMMA was dissolved by immersing PMMA/Gr/Target-Wafer into acetone, followed by thermal annealing at 400 °C in an inert atmosphere for 5 min, in order to remove the resist from the transferred graphene surface.

## Conclusions

In the present research, the relation between surface morphology, composition, and contact wetting angle of CVD grown SiO_2_ and Si_3_N_4_ surfaces, used as target wafers, were investigated. The exposure of SiO_2_/Si and Si_3_N_4_/Si surfaces to oxygen plasma revealed a decrease of C-contamination on the surface and the decrease of contact wetting angle down to ~ 2° for HDP, TEOS-SiO_2,_ and PE-Si_3_N_4_ films on Si wafers. Due to the increase of surface hydrophilicity, the large-scale graphene transfer was improved, resulting in homogenous graphene flakes (from 4 × 8 cm till 13 × 13 cm size) transferred on 200 mm HDP-, TEOS-SiO_2_/Si and PE-Si_3_N_4_/Si target wafers for reliable and reproducible graphene integration in Si-based technology platform.

## Supplementary Information

Supplementary Information.
