# Author Correction: Influence of plasma treatment on SiO_2_/Si and Si_3_N_4_/Si substrates for large-scale transfer of graphene

**DOI:** 10.1038/s41598-021-96605-z

**Published:** 2021-08-20

**Authors:** R. Lukose, M. Lisker, F. Akhtar, M. Fraschke, T. Grabolla, A. Mai, M. Lukosius

**Affiliations:** 1 IHP- Leibniz Institut für innovative Mikroelektronik, Im Technologiepark 25, 15236 Frankfurt (Oder), Germany; 2 Technical University of Applied Science Wildau, Hochschulring 1, 15745 Wildau, Germany

Correction to: *Scientific Reports* 10.1038/s41598-021-92432-4, published online 23 June 2021

The original version of this Article omitted an affiliation for M. Lisker. The correct affiliations for M. Lisker are listed below:

IHP- Leibniz Institut für innovative Mikroelektronik, Im Technologiepark 25, 15236, Frankfurt (Oder), Germany

Technical University of Applied Science Wildau, Hochschulring 1, 15745, Wildau, Germany

The original Article and accompanying Supplementary Information file have been corrected.

