# Supplementary material for: Influence of plasma treatment on SiO2/Si and Si3N4/Si substrates for large-scale transfer of graphene
Source: Sci Rep. 2021 Jun 23;11:13111. doi: 10.1038/s41598-021-92432-4 (PMC8222355; doi:10.1038/s41598-021-92432-4)
Supplement: Supplementary file 1 — Supplementary Information. [file 41598_2021_92432_MOESM1_ESM.docx]

**Influence of Plasma Treatment on SiO_2_/Si and Si_3_N_4_/Si Substrates for Large-Scale Transfer of Graphene**

R. Lukose^1*^, M. Lisker^1,2^, F. Akhtar^1^, M. Fraschke^1^, T. Grabolla^1^, A. Mai^1,2^, M. Lukosius^1^.

^1^*IHP- Leibniz Institut für innovative Mikroelektronik, Im Technologiepark 25, 15236 Frankfurt (Oder), Germany*

^2^*Technical University of Applied Science Wildau, Hochschulring 1, 15745 Wildau, Germany*

In this supplement information (SI) file, the additional XPS spectra concerning the surface composition of as-grown HDP-, TEOS-, Thermal-SiO_2_, and PE-Si_3_N_4_ on 200 mm Si wafers, is introduced (Fig. S1). In Fig. S2, the XPS results of corresponding target surfaces, after plasma treatment, are presented. The characteristic N 1s spectra of PE-Si_3_N_4_ is shown in Fig. S3. The possible cross-contamination through catalytic wafer (Ge/Si), electrolyte (NaOH) used for graphene transfer procedure, and post-transfer annealing for PMMA removal were neglected since no corresponding peaks related to Na 1s, N 1s, and Ge 2p were detected by the XPS method (Fig. S4).

**

**

**Figure S1.** The XPS spectra for as-grown films **(a)** C1s spectra, **(b)** O1s spectra, **(b inset)** Si 2p spectra for HDP-SiO_2_; **(c)** C1s spectra **(d)** O1s spectra **(d inset)** Si 2p spectra for TEOS-SiO_2_; and **(e)** C1s spectra **(f)** O1s spectra, **(f inset)** Si 2p spectra for thermal-SiO_2_.





**Figure S2.** The XPS spectra for plasma treated films **(a)** C1s spectra, **(b)** O1s spectra, **(b inset)** Si 2p spectra for HDP-SiO_2_; **(c)** C1s spectra, **(d)** O1s spectra, **(d inset)** Si 2p spectra for TEOS-SiO_2_; and **(e)** C1s spectra **(f)** O1s spectra, **(f inset)** Si 2p spectra for thermal-SiO_2_.


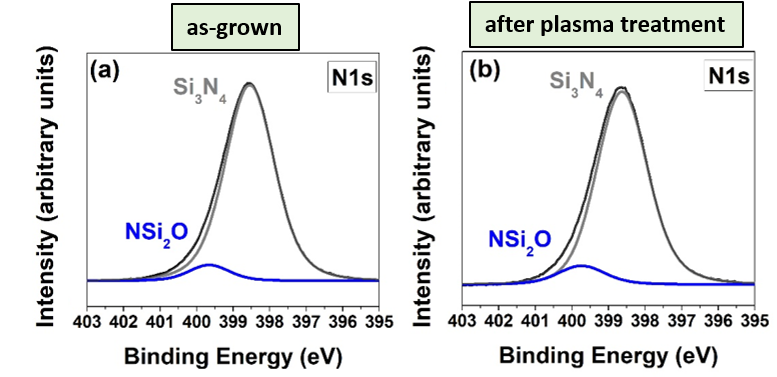


**Figure S3.** The characteristic XPS spectra of N 1s for as-grown (a) and plasma treated (b) Si_3_N_4_/Si surfaces.

**
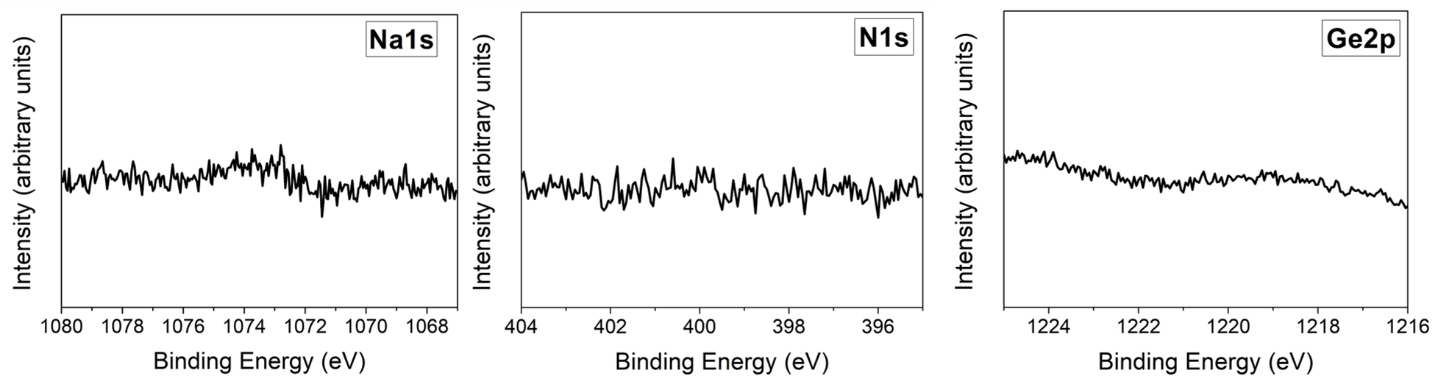
**

**Figure S4.** The XPS spectra example showing no characteristic Na 1s, N 1s, and Ge 2p peaks

**
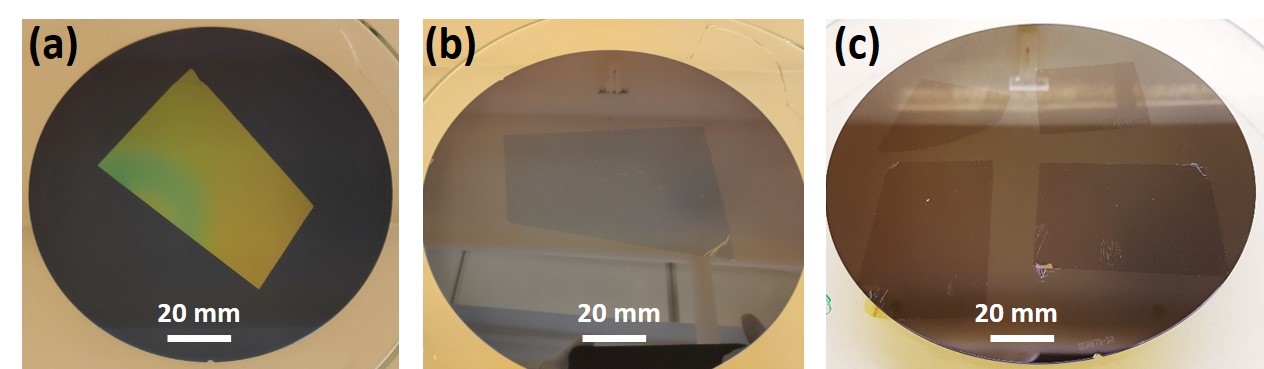
**

**Figure S5. (a)** The PMMA/Gr stack transferred on TEOS-SiO_2_; **(b)** Gr flake transferred on TEOS-SiO_2_ after the remove of PMMA; **(c)** Gr flake transferred on PE-Si_3_N_4_ after the remove of PMMA. The figures contains some artificial reflections.


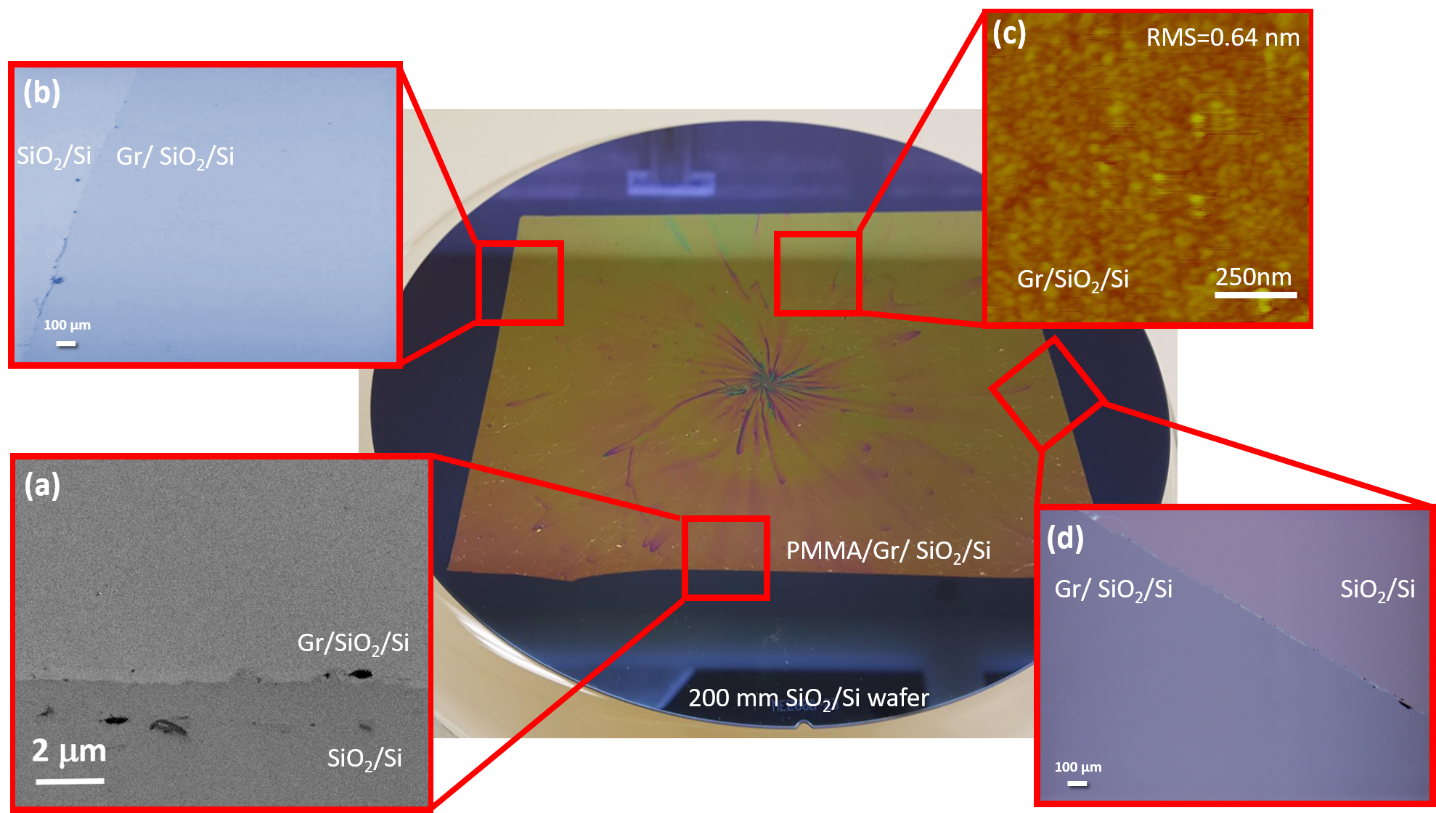


**Figure S6.** The main picture in the middle represents the transferred graphene flake with supporting PMMA layer for better contrast and visability. The zoomed figures represents transferred graphene without PMMA at different positions **(a)** scanning electron microscope at the flake edge; **(b)**, **(d)** optical microscope figures at different flake edges; **(c)** AFM figure with corresponding roughness of the transferred graphene.
